# Morphological plasticity of astroglia: Understanding synaptic microenvironment

**DOI:** 10.1002/glia.22821

**Published:** 2015-03-18

**Authors:** Janosch P. Heller, Dmitri A. Rusakov

**Affiliations:** ^1^ Department of Clinical and Experimental EpilepsyUCL Institute of NeurologyUniversity College LondonQueen SquareLondonUnited Kingdom

**Keywords:** astrocyte plasticity, perisynaptic astrocytic processes, super‐resolution microscopy

## Abstract

Memory formation in the brain is thought to rely on the remodeling of synaptic connections which eventually results in neural network rewiring. This remodeling is likely to involve ultrathin astroglial protrusions which often occur in the immediate vicinity of excitatory synapses. The phenomenology, cellular mechanisms, and causal relationships of such astroglial restructuring remain, however, poorly understood. This is in large part because monitoring and probing of the underpinning molecular machinery on the scale of nanoscopic astroglial compartments remains a challenge. Here we briefly summarize the current knowledge regarding the cellular organisation of astroglia in the synaptic microenvironment and discuss molecular mechanisms potentially involved in use‐dependent astroglial morphogenesis. We also discuss recent observations concerning morphological astroglial plasticity, the respective monitoring methods, and some of the newly emerging techniques that might help with conceptual advances in the area. GLIA 2015;63:2133–2151

## Introduction

Much to the surprise of many classically trained neurophysiologists, over the past few decades experimental evidence has emerged implicating electrically passive astroglia in rapid information transfer and possibly memory trace formation in the brain (Fields et al., 2014; Weaver, 2012). This line of thought has not been without controversy, however, mainly because our knowledge about the fundamentals of astroglial structure and physiology has fallen far behind what we know about nerve cells and their networks. Unlike neurons, astrocytes tend to have a sponge‐like morphology and occupy adjacent yet nonoverlapping tissue domains throughout the synaptic neuropil. In many instances, excitatory synapses are approached or surrounded by fine astrocytic protrusions, often termed perisynaptic astrocytic processes (PAPs). Although PAPs can be found at around 60% of synapses in the brain, the extent to which they enwrap synaptic structures can vary widely. Experimental evidence has been emerging suggesting that the synaptic astroglial coverage is dynamic and can be influenced by the animal's physiological state, local neuronal activity, induction of synaptic plasticity, or by certain behaviour.

Because structural remodeling of synapses is thought to be a basis for learning and long‐term memory formation in the brain (Chklovskii et al., 2004; Kwon and Sabatini, 2011), the use‐dependent morphological plasticity of PAPs is of special interest. However, the cellular architectural basis and the molecular machinery that are causal to physiological and morphogenetic events inside PAPs remain an enigma. It appears that the main reason for this uncertainty is the extraordinary small size of PAPs which is not only prohibitive for direct experimental probing but also imposes significant spatial restrictions on the local intracellular organization involved. Experimental advances in this direction are only beginning to emerge. The present review will discuss the up‐to‐date observations and concepts focusing on microscopic, sometimes nanoscopic, organization of astroglia, particularly in the vicinity of excitatory synapses. We will survey candidate molecular signaling mechanisms that could trigger and support astroglial morphogenesis on the small scale. A brief analysis will be given to the technical limitations in monitoring the reshaping of ultrathin astroglial structures such as PAPs. Finally, we will discuss novel methodological developments that should help researchers to gain previously unattainable insights into the fine molecular architecture of astrocytes.

## Molecular Makeup of Perisynaptic Astroglial Processes: Major Players

**Figure 1:**
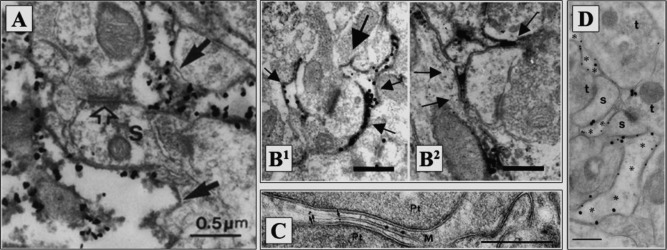
Astrocytes express various receptor types in their perisynaptic processes. (**A**) Silver‐enhanced (small grains) glutamine synthetase‐positive glial processes in the stratum lacunosum‐moleculare of CA1 region of the hippocampus. Finger‐like extensions of the glial processes at both sides of the spine (arrows) isolating these synaptic structures from the surrounding neuropil. s: spine. Adapted from (Derouiche and Frotscher, 1991). (**B**) Extremely fine PAPs (<100 nm, small arrows) ensheating pre‐ and/or postsynaptic elements (bold arrow in B1 pointing at synaptic cleft); mGluR2/3 (B1) and mGluR5 (B2) are present close to the synapse. Adapted from (Lavialle et al., 2011). (**C**) Double‐immunogold labeling of Kir4.1 (small particles, arrows) and aquaporin 4 (large particles) in vitreal perisynaptic Müller cell membranes (denoted M) surrounding photoreceptor terminals (Pt). Adapted from (Nagelhus et al., 1999). (**D**) Double labeling of astrocytic processes demonstrating co‐localization of GLT‐1 (small particles) and GLAST (large particles) (*) in rat hippocampus. S: dendritic spines; t: nerve terminals. Adapted from (Haugeto et al., 1996). Scale bars: 0.5 µm (B), 0.5 µm (C), 0.3 µm (D).

Widely considered as an active partner of the tripartite synapse (Haydon, 2001; Perea et al., 2009), astroglial protrusions including PAPs seem to express a battery of proteins that are important for maintaining efficient synaptic transmission (Dityatev and Rusakov, 2011; Halassa et al., 2007a). Among these, immunoelectron microscopy (EM) and related methods have identified glutamine synthetase (Fig. 1A) (Derouiche and Frotscher, 1991), metabotropic glutamate receptors (mGluRs) (Fig. 1B) (Arizono et al., 2012; Lavialle et al., 2011; Tamaru et al., 2001), aquaporins (Fig. 1C) (Nagelhus et al., 2004; Thrane et al., 2011), inwardly rectifying potassium channels Kir4.1 (Fig. 1C) (Higashi et al., 2001; Nagelhus et al., 2004), glutamate transporters (Fig. 1D) (Chaudhry et al., 1995; Haugeto et al., 1996; Lehre and Danbolt, 1998), metabotropic gamma‐aminobutyric acid (GABA)_B_ receptors (Charles et al., 2003), actin‐binding proteins (Lavialle et al., 2011; Molotkov et al., 2013), and cell adhesion molecules (Theodosis, 2002). Powerful, high‐affinity glutamate uptake (Danbolt, 2001), extracellular potassium buffering, and managing energy supply to neurons have been long‐established, classical functions of astroglia (Kimelberg, 2010; Newman, 1993; Suzuki et al., 2011) which are likely to occur in the synaptic proximity. More recently, it has been shown that astrocytes release a number of signaling molecules (gliotransmitters) such as glutamate (Bezzi et al., 2004), d‐serine (Henneberger et al., 2010; Panatier et al., 2006), adenosine triphosphate (ATP) (Cao et al., 2013), and tumour necrosis factor (TNF)‐α (Stellwagen and Malenka, 2006), which could in some cases exert regulatory influences on the nearby synapses. The molecular organization of the release machinery involved, however, remains unclear.

EM studies employing three‐dimensional reconstruction techniques have identified PAPs around most synaptic types, in various brain regions (see below). Their leaf‐like, ultrathin shapes correspond to a large surface‐to‐volume ratio thus suggesting the propensity to local chemical compartmentalization (Rusakov et al., 2011). It appears that in most cases, mitochondria, microtubules and endoplasmic reticulum are virtually absent from PAPs (Bernardinelli et al., 2014a; Reichenbach et al., 2010). Recently, quantitative three‐dimensional EM data have suggested that cellular organelles associated with Ca^2+^ signaling indeed tend to occur inside astroglia at some distance from the nearby excitatory synapses (Patrushev et al., 2013). However, PAPs do seem to contain individual ribosomes and glycogen granules, together with actin and actin binding proteins (Bernardinelli et al., 2014a; Derouiche and Frotscher, 2001; Fiala et al., 2003; Molotkov et al., 2013; Safavi‐Abbasi et al., 2001; Seidel et al., 1995). Moreover, recent results illustrate that despite general believe, fine astrocyte processes do contain some types of calcium stores and sinks as seen with EM (Lovatt et al., 2007; Sahlender et al., 2014; Volterra et al., 2014).

## Subtypes and Brain‐Region Specificity of Astroglia

**Figure 2:**
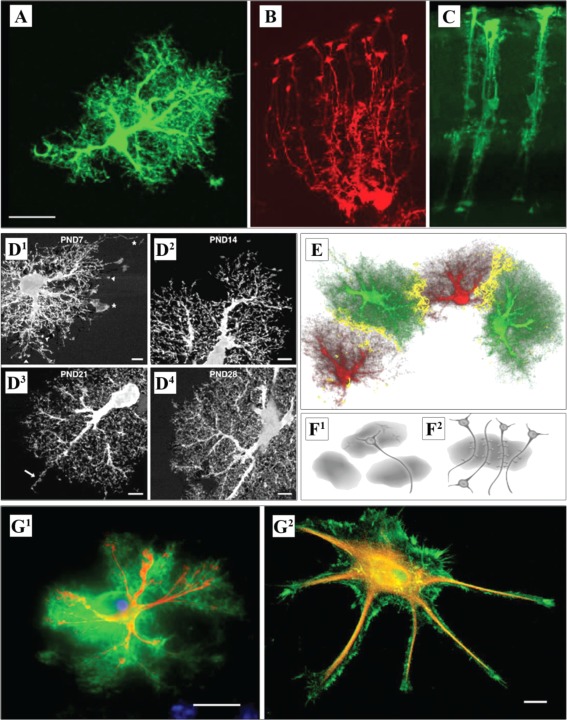
Varied morphology of astrocyte types. (**A**) Protoplasmic astrocyte in the mouse neocortex with numerous processes. (**B**) Bergmann glial cells from the mouse cerebellum. Note cell bodies are localized at one end, and distal process endings contacting the pia mater. (**C**) Müller cells of the mouse retina span the entire distance between the vitreous body and the retinal pigment epithelium. (A–C) adapted from (Reichenbach et al., 2010). (**D**) The degree of ramification of protoplasmic astrocytes at PND7 (D1), PND14 (D2), PND 21 (D3), and at 1 month (D4). Adapted from (Bushong et al., 2004). (**E**) Three‐dimensional reconstruction of astrocytes in the dentate gyrus. The yellow zone shows the border area where cellular processes of two adjacent astrocytes interdigitate. Adapted from (Wilhelmsson et al., 2006). (**F**) Schematic representation of functional synaptic islands. Different neuronal compartments can potentially be modulated by different astrocytes (F1). A group of dendrites from several neurons are enwrapped by a single astrocyte. Synapses localized within the territory of this astrocyte could potentially be affected in a coordinated manner by this glial cell (F2). Adapted from (Halassa et al., 2007b). (**G**) Double labeling of an acutely isolated (G1) and a cultured (G2) astrocyte for GFAP (red) and ezrin (green). Adapted from (Derouiche and Frotscher, 2001; Reichenbach et al., 2010). Scale bars: 20 µm (A), 5 µm (D), 15 µm (G1), 10 µm (G2).

In the rodent brain one can distinguish two main types of astrocytes: fibrous and protoplasmic (Gallo and Deneen, 2014; Rodnight and Gottfried, 2013). The fibrous type cells occur in the white matter and are thought to promote myelination of axons through interaction with oligodendrocytes (Lundgaard et al., 2014; Molofsky et al., 2012). Protoplasmic astroglia are found in the synaptic neuropil; the present review is primarily focused on this astrocyte type (Fig. 2A). In addition to these two large classes of astrocytes, two additional subtypes have been identified in the brain of primates: interlaminar and varicose projection astrocytes (Colombo and Reisin, 2004; Colombo et al., 1995; Oberheim et al., 2009; Sosunov et al., 2014). Interlaminar astrocytes are found in the upper cortical layers from which they extend long glial fibrillary acidic protein (GFAP)‐positive processes through layers 2–4 (Colombo and Reisin, 2004; Colombo et al., 1995; Oberheim et al., 2009). The recently identified varicose projection astrocytes are located in cortical layers 5 and 6 of higher primates (Oberheim et al., 2009). They extend shorter spiny processes and one to five long projection fibers with regularly spaced varicosities within the deeper layers of the cortex that can transverse several protoplasmic astrocyte islands (Oberheim et al., 2009). Furthermore, specialized astrocytic cells exist in the cerebellum (Bergmann glia) (Fig. 2B) and in the retina (Müller glia) (Fig. 2C) (Eroglu and Barres, 2010). In fact, a combination of labeling strategies has suggested no less than nine different astrocyte classes in the adult mouse CNS: fibrous and protoplasmic astrocytes, Bergmann glia, ependymal glia, marginal glia, perivascular glia, radial glia, velate glia, and tanycytes (Emsley and Macklis, 2006). The authors described that the classes of astrocytes appeared in different proportions in different brain regions, suggesting various within‐group heterogeneities (Emsley and Macklis, 2006; Oberheim et al., 2012).

Interestingly, the astroglial receptor expression pattern seems to be related to the brain region in which the respective receptors or their transmitters are well represented across the local neuronal population (Verkhratsky and Kettenmann, 1996; Verkhratsky et al., 1998). There is no surprise therefore that the distribution of astroglial receptor or transporter subtypes varies across brain areas. For example, Bergmann glia in the cerebellum exhibit high levels of GLAST (EAAT1) and almost no GLT‐1 (EAAT2). In contrast, astrocytes in the hippocampus have very high levels of GLT‐1 and hardly any GLAST (Chaudhry et al., 1995; Furuta et al., 1997; Tzingounis and Wadiche, 2007). Furthermore, expression of glutamate transporters seems to depend on neuronal activity: decreased synaptic activity appears to downregulate levels (Benediktsson et al., 2012; Yang et al., 2009). There seems to be further differentiation of astrocytes within regions. For instance, it has been reported that in the hippocampus there are two populations of astrocytes, one expressing glutamate transporters and the other mainly α‐amino‐3‐hydroxy‐5‐methyl‐4‐isoxazolepropionic acid receptors (AMPARs) instead (the latter representing mainly NG2 proteoglycan expressing cells) (Matthias et al., 2003). Furthermore, a study analyzing the gene expression profile of cortical mouse astrocytes identified two groups of GLT‐1 and aquaporin 4‐expressing cells depending on whether they express GFAP or not (Lovatt et al., 2007). These cell populations differed in ∼1% of their expression profiles (Lovatt et al., 2007). On the other hand, the expression of potassium channels varies among brain regions although the main astroglial channel Kir4.1 seems to be widely expressed across the CNS (Butt and Kalsi, 2006). The appearance and identity of astroglia, however, may also change with development.

## Formation of Astroglial Architecture During Development

Both neurons and glia originate from the neural precursor cells (radial glial cells) (Freeman, 2010; Kanski et al., 2014). During development, neurons appear first, followed by glia, with numerous extrinsic and intrinsic factors involved in the cell generation transition (Freeman, 2010). Notably, astrocyte development depends on Wnt and JAK‐STAT signaling cascades (Freeman, 2010; Kanski et al., 2014; Namihira and Nakashima, 2013). In addition to radial glia cell division, astrocytes can arise from clonal division of early differentiated astrocytes (Ge et al., 2012; Sloan and Barres, 2014). The latter mechanism might contribute to the specialization of astrocyte domains among and within brain regions (Garcia‐Marques and Lopez‐Mascaraque, 2013). The known pathways of astrocyte generation are far from exhaustive, with the possibility remaining of a still unknown astrocyte‐restricted progenitor population (Sloan and Barres, 2014).

**Figure 3:**
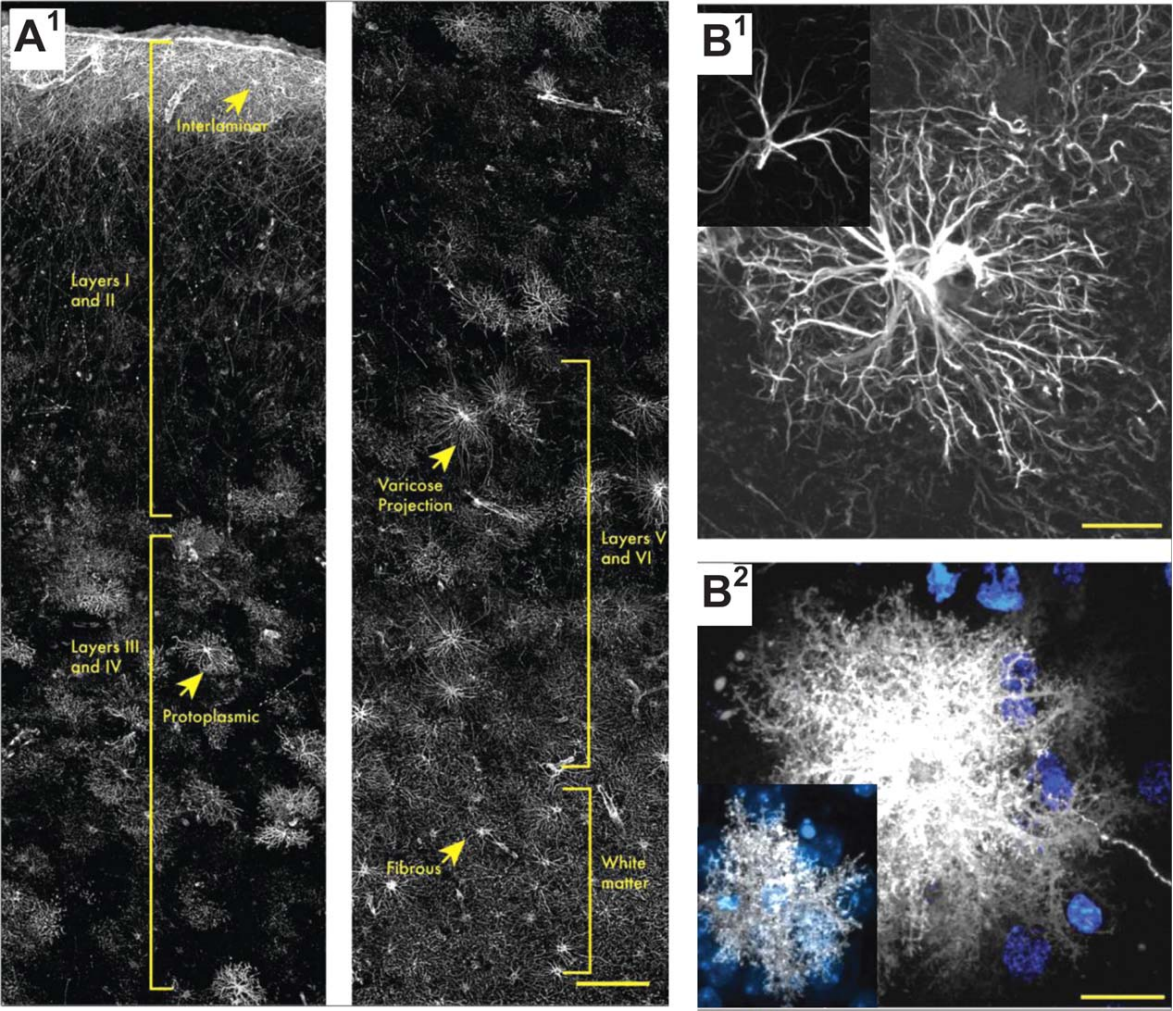
Morphological diversity of astroglia within and among species. (**A**) Laminar astrocytes are present in human cortical layer 1, and the cells extend long processes through layers 2 to 4. Protoplasmic astrocytes can be found in layers 2 to 6. Varicose projection astrocytes are located in layers 5 and 6 from where they extend mm‐long processes with regularly spaced varicosities. Fibrous astrocytes reside in the white matter. (**B1**) Main processes of mouse protoplasmic astrocytes (inset) are less extended and developed than human astroglia (main image). (**B2)** Accordingly, fine processes and the entire morphology of astrocytes in mice correspond to much smaller functional islands (inset) compared with human astroglia (main image). Scale bars: 150 µm (A), 20 µm (B); Adapted from (Oberheim et al., 2009).

In rats, astrocytes start extending fine filopodia for up to postnatal day 14 (PND14). By PND21, astroglia in the brain develop highly ramified processes (Bushong et al., 2004; Parnavelas et al., 1983). In hippocampal area CA1, astrocytes develop a homogenous morphology by PND14 (compared with PND7; Fig. 2D) although astrogliogenesis is still ongoing (Bushong et al., 2004). While developing, astrocytes extend longer and less ramified processes first (Fig. 2D1), with the formation of the spongiform processes starting at the soma and extending centrifugally until all the processes are equipped with fine protrusions. At this stage, neighbouring astrocytes “meet,” and the inter‐domain boundaries are formed (Fig. 2D2–D4) (Bushong et al., 2004). Whether all primary processes become ramified or whether some are eliminated during development remains poorly understood. Nonoverlapping astrocyte domains are formed at around one month of age (Fig. 2E) (Bushong et al., 2004; Halassa et al., 2007b), with one astrocyte approaching processes of many neurons and one neuron crossing territories of many astrocytes (Fig. 2F) (Halassa et al., 2007b). As a result of various mechanisms affecting astrocyte formation during development, by the adulthood various types of astroglia could be found, in relative proximity from one another, across different cortical layers (Fig. 3A).

## Developmental Changes in the Molecular Makeup of Astrocytes

In parallel with morphology, the astroglia gene expression profile, hence their protein makeup, also changes during development. In mice, genes become upregulated encoding proteins involved in amino acid synthesis and degradation, and also in phagocytosis (Cahoy et al., 2008). Furthermore, genes that are important in actin cytoskeleton signaling become upregulated (Cahoy et al., 2008), which suggests the importance of this cytoskeletal molecular cascade in PAP motility (see below). However, gene expression data provide little clues regarding the intracellular distribution of proteins.

Glutamate uptake is an essential astroglial function, and the way glutamate is cleared from the perisynaptic space changes in the first postnatal days, from mainly diffusion escape (through large interstitial gaps characteristic for early development) to high‐affinity uptake by astrocytes (Thomas et al., 2011). Correspondingly, the expression of astroglial glutamate transporters increases, with a gradual shift from predominantly GLAST to GLT‐1 during maturation (Furuta et al., 1997; Regan et al., 2007). The expression of astroglial glutamate receptors is also developmentally controlled. Single‐cell gene expression assays have indicated that the expression of kainate receptors and AMPARs declines throughout development (Rusnakova et al., 2013). The genes encoding *N*‐methyl‐d‐aspartate receptor (NMDAR) subunits also change their expression levels even though NMDAR appears to dominate ionotropic glutamate receptor types in adult mouse astroglia. Metabotropic glutamate receptor mGluR5 is widely present in young animals but its expression declines with age while the mGluR3 subtype becomes dominant in adult mice and in humans (Cai et al., 2000; Rusnakova et al., 2013; Sun et al., 2013). Electrophysiological evidence suggests that during development and aging (from 1 to 21 months) the functional AMPAR expression in astrocytic membranes progressively declines whereas the currents mediated by P2X and NMDA receptors first increase towards adulthood and then decrease in older mice (Lalo et al., 2011).

Development and maturation also affect astroglial mechanisms involved in water balance and (functionally related) potassium buffering. Aquaporin 4 appears after PND20 and its levels increase afterwards, according to single‐cell gene expression data (Rusnakova et al., 2013). The major potassium channel Kir4.1 is downregulated to its stable level in the hippocampus within the first ten postnatal days (Seifert et al., 2009). In contrast to most astrocytic receptors, the levels of GFAP as well as of the calcium binding protein s100β increase during aging (Nichols, 1999; Sheng et al., 1996). In accord with these observations, a global increase in GFAP expression in the aged brain is detectable, suggesting an increased number of astrocytes in older brains (Cotrina and Nedergaard, 2002). Alternatively, because only ∼15% of astrocytes stain for GFAP in the mouse brain (Bushong et al., 2002), global increases in GFAP labeling could also suggest that various astrocyte subtypes start expressing GFAP during aging. It also appears that the interaction of astrocytes with nearby neurons changes with age. In simple terms, neurons seem to require direct contact with astrocytes for synaptogenesis during early development whereas secreted factors become more important during aging (Allen, 2013) (see below).

## Human Brain Astrocytes

Importantly, there exist several visible differences between rodent and human astrocytes (Fig. 3B). First, the ratio of glia to neurons in the human cortex is ∼1.65:1. In rodents however, this ratio is close to 0.3:1 (Nedergaard et al., 2003; Sherwood et al., 2006). The human brain appears to exhibit classes of GFAP‐positive cells that are different from cells found in the rodent brain. For instance, in addition to protoplasmic and fibrous astrocytes, interlaminar and varicose projection astrocytes are only found in primates (Colombo and Reisin, 2004; Colombo et al., 1995; Oberheim et al., 2009; Sosunov et al., 2014). Furthermore, human GFAP‐positive protoplasmic astrocytes are larger and more complex than rodent cells (Fig. 3B), they exhibit enhanced calcium responses, increased calcium wave velocities, and a greater overlap between neighbours (Oberheim et al., 2009; Sosunov et al., 2014). Human and rodent astrocytes also differ with regards to their transcripts (Miller et al., 2010) as do in fact astrocytes from different brain regions (Doyle et al., 2008; Rodriguez et al., 2014; Yeh et al., 2009). Nonetheless, experiments in acute slices from the human cortex and hippocampus have documented astroglial Ca^2+^ signals in response to excitatory afferent stimulation or application of glutamate, cannabinoid, and purinergic receptor agonists (Navarrete et al., 2013). Furthermore, the gene expression patterns for major glutamate transporters and several subtypes of mGluRs appear generally compatible between human and mouse adult brain astrocytes (Sun et al., 2013). Interestingly, a recent study found that human astrocytes engrafted in the rodent brain maintain their “primate” features (Han et al., 2013). The grafted cells appear to boost basal excitatory synaptic transmission and its potentiation, show enhanced levels of TNF‐α, and help the chimeric animals to perform better in some specific memory tasks (Han et al., 2013; Windrem et al., 2014). This was an important observation, especially because astrocyte morphology and function appear somewhat evolutionarily conserved: in *Drosophila*, astrocytes also form a dense meshwork of processes that wrap around synapses and are essential for animal development and survival (Doherty et al., 2009; Stork et al., 2014). Such observations suggest a reasonable scope for the extrapolation of astroglial physiology from experimental animals (mainly rodents) to humans.

## Astroglial Coverage of Synapses

In rodents, synaptic neuropil contains on average 1.5 to 2.0 synapses per μm^3^ (Rusakov et al., 1998), whereas in humans this value is slightly lower, ∼1.1 (DeFelipe et al., 2002). Depending on the brain region, the tissue volume occupied by one (nonoverlapping) astrocyte in rodents varies between 20,000 and 80,000 µm^3^ (Bushong et al., 2002; Halassa and Haydon, 2010; Oberheim et al., 2012), and 300 to 600 dendrites of principal neurons normally approach a single astrocyte (Halassa and Haydon, 2010). These relationships indicate that a single astrocyte approaches 20 to 120 thousand synapses in the rodent brain, and ∼270 thousand to 2 million synapses in the human brain (Bushong et al., 2002; Oberheim et al., 2009, 2006).

**Figure 4:**
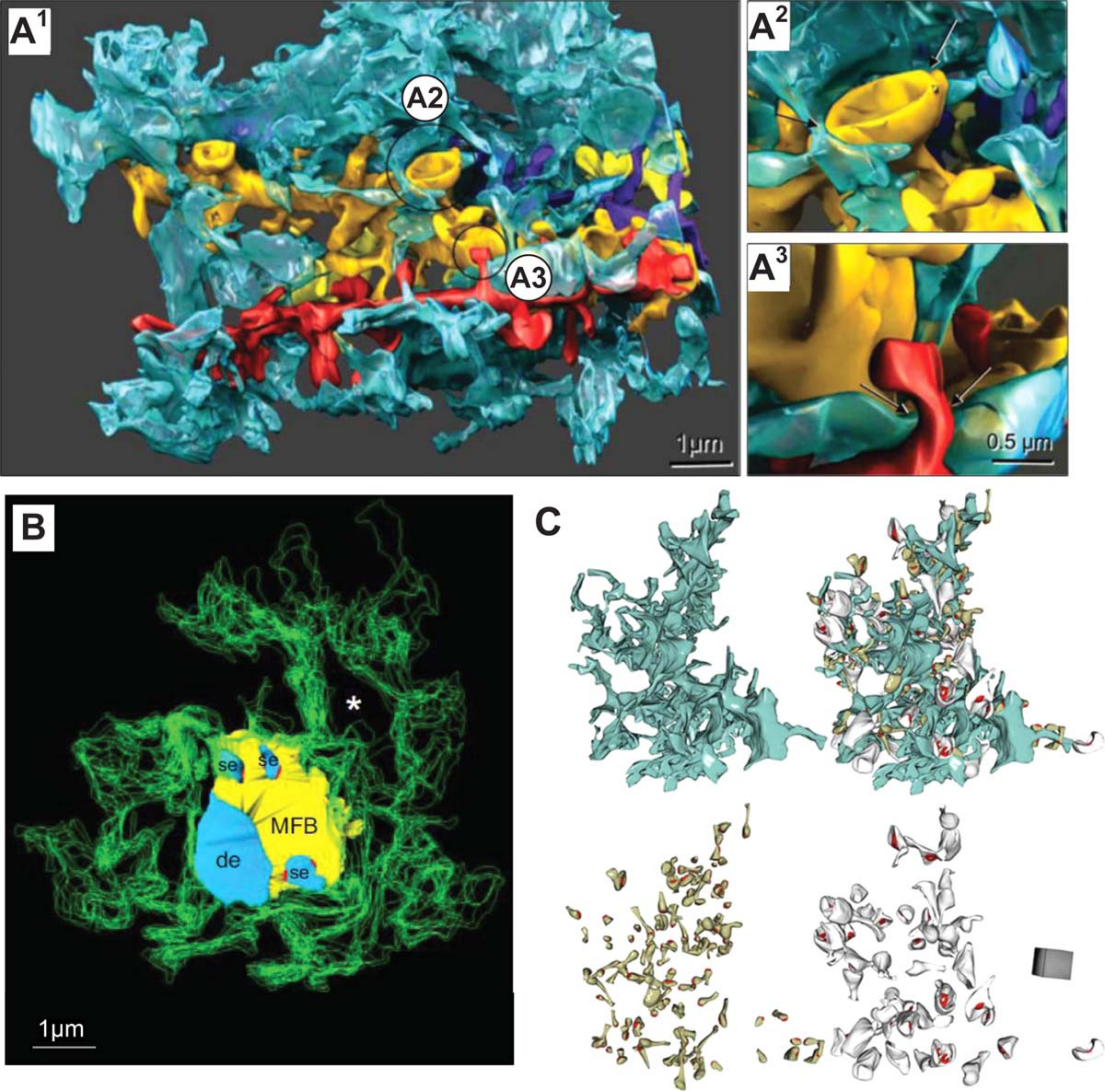
Astroglial coverage of excitatory synapses in three hippocampal areas. (**A1**) Three‐dimensional EM reconstruction of a single astroglial process (blue) in contact with four reconstructed dendrites (gold, yellow, red, purple) in hippocampal area CA1. (**A2**) Mushroom spine example with ∼50% circumvent contacted by astroglia (arrows). (**A3**) Thin dendritic spine example, with its neck adjacent to astroglia (arrows). Scale bar in A3 applies to A2‐3. Adapted from (Witcher et al., 2007). (**B**) Three‐dimensional EM reconstruction showing the astrocytic processes (green outlines) that surround a giant mossy fiber synapse in hippocampal area CA3, including the axonal bouton (yellow), the postsynaptic dendrite (de), and postsynaptic spiny excrescences (se, light blue). Fine astrocytic protrusions cover presynaptic and postsynaptic structures but do not reach synaptic active zones (red) on spiny excrescences; asterisk (void), location of an adjacent axonal bouton (not shown). Adapted from (Rollenhagen et al., 2007). (**C**) Three‐dimensional reconstruction of an astrocyte fragment (blue) shown with and without adjacent thin (left, grey) and mushroom (right, dark yellow) dendritic spines equipped with PSDs (red). Adapted from (Medvedev et al., 2014).

The actual three‐dimensional distances between astrocytic membranes and dendritic spines can vary from the immediate contact to hundreds of nanometers (Lushnikova et al., 2009; Medvedev et al., 2014; Patrushev et al., 2013; Spacek and Harris, 1998). Although PAPs can be found in all brain regions, only a proportion of synapses are actually in immediate contact with them. Moreover, this proportion differs across the brain regions. In the rat neocortex only 29 to 56% of excitatory synapses are enwrapped by astrocytic processes, with variable extent of the immediate contact (Bernardinelli et al., 2014a; Reichenbach et al., 2010). In contrast, in layer IV of the somatosensory cortex in adult mice 90% of spines are in contact with astrocytes (Bernardinelli et al., 2014a) whereas in the rat hippocampus this number is ∼62 to 90% (Ventura and Harris, 1999; Witcher et al., 2007). It appears that astrocytic processes approach preferentially synapses that have complex (e.g. perforated) postsynaptic densities (PSDs) (Fig. 4A) (Witcher et al., 2007) and tend to occur on the postsynaptic side (Lehre and Rusakov, 2002). In contrast, in the hippocampal CA3 region PAPs virtually isolate synapses from the surrounding tissue and hence make spillover almost impossible (Fig. 4B) (Rollenhagen et al., 2007).

An association between astroglial coverage and synapse identity has been suggested by the current data indicating that astroglia in the hippocampal dentate tend to cover synapses hosted by thin dendritic spines (related to NMDAR‐only synapses (Matsuzaki et al., 2001)) to a greater extent compared with synapses on thicker spines (Fig. 4C) (Medvedev et al., 2014). In organotypic slices of rat hippocampus, 85% of spine synapses are enwrapped by PAPs; 97% of synapses with complex PSDs exhibit some astrocyte coverage but only 78% of simple synapses (Lushnikova et al., 2009). Recent data suggest, however, that in the CA1 region, astrocytic processes hardly ever cover the axon‐spine interface and hence should allow for extrasynaptic escape of glutamate (Bernardinelli et al., 2014a). In contrast to individual excitatory synapses that are often approached by astroglia, PAPs are usually not present within synaptic glomeruli, groups of functionally related synapses found within the sensory thalamus, the olfactory bulb and the cerebellar cortex (Reichenbach et al., 2010). Thus, by surrounding individual glomeruli astroglia might contribute to forming distinct functional neuronal units (Reichenbach et al., 2010). In the cerebellar glomeruli, in which mossy fibers synapse on granule cells, only ∼15% of synaptic terminals are in contact with PAPs, favoring neurotransmitter spillover within the glomerulus (Mitchell and Silver, 2000; Sylantyev et al., 2013; Xu‐Friedman et al., 2001). On the other hand, synapses on Purkinje cells are isolated to a higher degree through Bergmann glia (Xu‐Friedman et al., 2001). The synapses with the closest contact to glial cells in the cerebellum are formed by climbing fibers, with ∼87% of synapse area being covered. Parallel fiber synapses are also in relatively close contact with Bergmann glia, with ∼65% of synapse area covered (Xu‐Friedman et al., 2001). It is clear, however, that reliable estimation of synaptic astroglial coverage could be difficult: firstly, because of the uncertainties arising from the highly complex three‐dimensional geometry of the synapse—PAP juxtaposition, and, secondly, because of the errors inherent to the three‐dimensional reconstruction (or visualization) methods employed.

## Current Methods to Monitor Fine Astrocyte Morphology: Promise and Challenge

Protoplasmic astrocytes feature thousands of fine protrusions that can easily be distinguished from the larger, GFAP‐positive primary and secondary branches visible with conventional microscopy (Fig. 3B). The GFAP‐positive main branches of an astrocyte make up only ∼15% of its total volume (Bushong et al., 2002), thus leaving the bulk of astroglial morphology largely beyond the diffraction limit of conventional optical microscopy. Structural changes in astroglial filopodia‐like protrusions can be routinely observed using time‐lapse light microscopy in cultured hippocampal or cortical astrocytes (Cornell‐Bell et al., 1990a; Lavialle et al., 2011; Molotkov et al., 2013). In acute brain slices some mobile astrocytic processes can be detected (Hirrlinger et al., 2004; Nishida and Okabe, 2007; Nixdorf‐Bergweiler et al., 1994). Very fine, and apparently highly mobile, astrocytic processes have been reported in organotypic brain slices using a membrane‐bound GFP (Benediktsson et al., 2005). Furthermore, co‐labeling of dendritic spines and PAPs in hippocampal (Halassa et al., 2007a; Perez‐Alvarez et al., 2014; Verbich et al., 2012) and cerebellar slices (Lippman Bell et al., 2010; Lippman et al., 2008) as well as *in vivo* (Bernardinelli et al., 2014b; Perez‐Alvarez et al., 2014) suggested that PAPs are highly dynamic structures that engage and disengage with dendritic spines, also responding to the induction of synaptic plasticity (Bernardinelli et al., 2014b; Perez‐Alvarez et al., 2014).

In this context, it is important to point out that monitoring fluorescence‐labeled morphology of ultrathin astroglial structures is prone to ambiguities. Firstly, relatively small fluctuations in imaging conditions, dye concentration or focal plane position could be mistaken for genuine structural changes. Secondly, applying brightness threshold or other filtering techniques to the fluorescent signal generated on the nanoscale often leads to misrepresentation. For instance, a relatively thick cellular process first seen in the microscope as a bright spot could appear shrunk (or disappear from view altogether) when in fact it simply transforms into a larger and thinner leaf‐like structure: the latter might appear as shrinkage simply because fluorescence generated by the ultrathin transformed structure may fall below the detection threshold. Finally, it might not always be possible to separate molecular re‐arrangement or movement of genetically encoded fluorescent markers (such as membrane‐bound proteins) within astroglial compartments from genuine morphological alterations. Therefore, caution should be exercised in optical monitoring studies to avoid registration of spurious morphological changes on the nanoscale.

EM does not have the advantage of monitoring live cells in real time but it has resolution sufficient to identify and document the thinnest astroglial protrusions. Ultrastructure analyses combined with other methods helped to demonstrate rapid and reversible structural remodeling of astrocytic processes in the hypothalamus (Becquet et al., 2008; Theodosis et al., 1981). These changes follow several types of stimulation, including lactation, parturition, and stress (Montagnese et al., 1988; Oliet et al., 2001; Salm, 2000; Theodosis, 2002). Similarly, in the visual cortex, structural changes in PAPs can be observed to parallel structural and functional synaptic plasticity (Jones and Greenough, 1996). Chronic whisker stimulation induced structural changes in PAPs enhancing synaptic PAP coverage, as well as local glutamate transporter expression, in the barrel cortex of adult mice (Genoud et al., 2006). Inducing long‐term potentiation (LTP) in adult rats *in vivo* also leads to PAP structural plasticity in the dentate gyrus of the hippocampus (Wenzel et al., 1991). In the amygdala, however, only ∼50% of synapses are contacted by astrocytic processes which cover only ∼20% of the synaptic circumvent (Ostroff et al., 2014). In this area, increasing synaptic strength leads to an increase in the number of synapses that are not associated with an astrocyte, whereas decreasing synaptic strength enhances the number of small synapses that are contacted by an astrocyte (Ostroff et al., 2014). In organotypic slices, LTP induction through theta‐burst stimulation of Shaffer collaterals increased glial coverage of pre‐ and postsynaptic elements in an NMDAR‐dependent manner (Lushnikova et al., 2009). Furthermore, kindling and ischemic preconditioning also induces the growth of astroglial processes (Hawrylak et al., 1993). Thus, a growing body of experimental evidence has been accumulated suggesting use‐dependent structural responsiveness of astroglial processes in the vicinity of excitatory synapses. Clearly, further reconciliation of high‐resolution EM observations in fixed tissue with optical recordings in live preparations will lead to a better understanding of the nanoscopic remodeling of synaptic microenvironment pertinent to such plasticity.

## Triggering Structural Plasticity of Astroglia by Excitatory Neuronal Activity

It is logical to think that use‐dependent remodeling of synaptic connections should involve alterations in the entire synaptic microenvironment (Caroni et al., 2012), which in many cases contains PAPs. Notwithstanding the aforementioned concerns about microscopic monitoring of nanostructures, candidate molecular mechanisms underlying this synapse‐astroglia relationship are beginning to emerge. A link between spine and PAP movement has been reported with confocal time‐lapse imaging: blocking inhibitory transmission to boost network activity did not enhance such movements (Haber et al., 2006). Conversely, blockade of sodium channels (and therefore action potential‐dependent transmission) with tetrodotoxin (TTX) did not reduce PAP motility (Verbich et al., 2012). In the same study, however, glutamate did induce PAP structural changes whereas the blockage of glutamate transporters or TTX application impeded PAP movements, suggesting that glutamate signaling could contribute to PAP mobility (Verbich et al., 2012).

In cultured astrocytes, the ionotropic glutamate receptor agonists glutamate, kainate, and quisqualate evoke filopodia outgrowth, and so do agonists of mGluRs (Cornell‐Bell et al., 1990b; Lavialle et al., 2011). However, selective NMDAR agonists do not appear to trigger morphological changes whereas mGluR antagonists block them (Cornell‐Bell et al., 1990b). Astrocytes (and probably PAPs) express both, AMPARs and mGluRs (see above) and both receptor types might be involved in astrocytic filopodia outgrowth (Bernardinelli et al., 2014a). More recently, the induction of LTP in hippocampal slices and whisker stimulation has led to measureable PAP displacement in the hippocampus and the barrel cortex, respectively (Bernardinelli et al., 2014b; Perez‐Alvarez et al., 2014). It was suggested that PAP motility is (a) synapse‐specific, (b) neuronal activity‐dependent (application of TTX blocks PAP movement), (c) mGluR‐dependent, (d) Ca^2+^‐dependent, and (e) IP_3_‐dependent (Bernardinelli et al., 2014b; Perez‐Alvarez et al., 2014). These observations raise an intriguing question of whether there are organizational principles for the presence and shape of PAPs depending on the brain area, synaptic types and species (Reichenbach et al., 2010).

Across astrocyte types, mGluR signaling through Gq proteins triggers calcium release from intracellular stores such as the endoplasmic reticulum (Volterra et al., 2014). This cascade in turn might influence actin dynamics and hence filopodia growth, as suggested in studies using calcium uncaging in astrocyte cultures and genetic attenuation of IP_3_ signaling *in vivo* (Molotkov et al., 2013; Tanaka et al., 2013). PAPs do contain actin as their cytoskeleton basis (Safavi‐Abbasi et al., 2001), and inhibition of actin polymerization disables PAP mobility (Haber et al., 2006). Astrocytic filopodia outgrowth does not require new synthesis or degradation but rather a redistribution of existing cytoskeletal proteins (Safavi‐Abbasi et al., 2001).

## Molecular Machineries of Astroglial Morphogenesis: Cytoskeleton, Growth Factor, Cell Adhesion

In cultures (Safavi‐Abbasi et al., 2001) as well as slices (Lippman et al., 2008; Nestor et al., 2007; Nishida and Okabe, 2007) astrocytic processes can adapt quickly to changes in their environment through a mechanism involving the GTPase Rac‐1. Moreover, expression of a dominant negative form of the actin‐binding protein profilin‐1 inhibited filopodia formation and motility (Molotkov et al., 2013). Also the actin‐binding proteins alpha‐actinin, alpha‐adducin, and ezrin have been localized in PAPs (Fig. 2G) (Lavialle et al., 2011; Safavi‐Abbasi et al., 2001; Seidel et al., 1995). Ezrin connects the plasma membrane to the actin cytoskeleton and can be regulated through phosphorylation/dephosphorylation and hence does not require protein synthesis or breakdown (Reichenbach et al., 2010). Therefore, knockdown of ezrin interfered with astrocytic and neuronal filopodia growth and movement (Lavialle et al., 2011; Matsumoto et al., 2014). Clearly, the cytoskeletal machinery is essential to at least some forms of structural astroglial plasticity.

Fibroblast growth factor (FGF) has been identified as a factor mediating morphological changes in astrocytes, by initiating outgrowth of highly ramified processes into the neuropil in *Drosophila* (Agarwal and Bergles, 2014; Stork et al., 2014). Also in stem cell‐derived spinal cord astrocytes, FGF exposure directs the maturation of the cells (Roybon et al., 2013). Furthermore, a study in mice showed that FGF8 increased the number and branching of processes in developing and cultured astrocytes (Kang et al., 2014). Whether this growth factor is actively secreted by neurons to attract PAPs remains to be determined.

Cell adhesion molecules appear to be another logical candidate device to mechanistically explain the underpinning of structural astroglial plasticity. One of the most studied adhesion molecules that could be involved in direct signaling between astrocytes and neurons has been EphA4 (Carmona et al., 2009; Filosa et al., 2009; Murai and Pasquale, 2004; Tremblay et al., 2009). This receptor tyrosine kinase is located in dendritic spines, and its ligand ephrin‐A3 is enriched in PAP membranes (Murai et al., 2003). Binding of the two molecules is important for normal spine morphology (Murai et al., 2003) and mitigating the contact leads to unstable spines (Nishida and Okabe, 2007). Furthermore, the binding of exogenous and endogenous ephrin‐A to astrocytic EphA ligands mediates astrocyte process outgrowth (Nestor et al., 2007). Moreover, ephrin signaling is involved in LTP as it regulates astroglial glutamate transporter expression (Filosa et al., 2009). In addition to ephrins, neuroligins might be involved in mediating neuron‐astrocyte communication and PAP outgrowth as astrocytes in neuroligin KO mice showed morphological aberrations (Cao et al., 2012). These mechanisms have been implicated when observing patients with autism, a disease linked to mutations in neuroligins and neurexins (Bernardinelli et al., 2014a; Chih et al., 2005). Other adhesive molecules that have been involved in neuron‐astrocyte binding and that might be involved in boosting PAP morphogenesis are Ng‐CAM (Grumet et al., 1984), SynCAM1 (Sandau et al., 2012), NCAM (Theodosis et al., 2004), and protocadherin‐gammaC5 (Garrett and Weiner, 2009; Li et al., 2010). However, whether and how these signaling molecules and their partners or ligands act within PAPs in organized tissue *in situ* remains to be established. It appears that advances in super‐resolution microscopy will play a major role (see below) in our understanding of the exact molecular make‐up of PAPs and its dynamic changes.

## Molecular Machineries of Astroglial Morphogenesis: Volume Control and Connexin Signaling

Three‐dimensional EM reconstructions of the CA1 neuropil have shown that the extracellular space (ECS) around axonal terminals, dendrites, and glial membranes is shaped nonuniformly featuring various tunnels and sheets (Kinney et al., 2013). It appears that astrocytes should be able to regulate extracellular (volume) communications by swelling or shrinking (Mongin and Kimelberg, 2005; Reichenbach et al., 2010) and thus changing the distribution of the tunnels and sheets within the ECS (Kinney et al., 2013). This volume control by astrocytes can be modulated by aquaporins and Kir4.1 that are expressed in PAP membranes and control swelling (Anderova et al., 2014; Nagelhus et al., 2004; Thrane et al., 2011).

In addition to astrocyte‐neuron relationship, the intercellular contacts in the astrocyte networks could be important for PAP plasticity. Astrocytes are connected through the gap junction molecules connexin 43 (Cx43) and Cx30 (Anders et al., 2014; Chever et al., 2014; Pannasch and Rouach, 2013; Pannasch et al., 2011). Gap junctions might be involved directly or indirectly in synapse‐glia signaling (Houades et al., 2008). Recently, it has been reported that Cx43 mediates synaptic efficacy by setting the levels of synaptic glutamate in mice (Chever et al., 2014). Interestingly, the loss of Cx30 has fundamental consequences on the structure of PAPs and on the strength of excitatory synapses. Astrocytes devoid of the gap junction protein invade the synaptic cleft, leading to decreased AMPAR‐mediated synaptic transmission as glutamate was taken up by the astrocytes before it reached the postsynapse (Pannasch et al., 2014). Moreover, astrocytes lacking Cx43, or Cx30 and Cx43 (Chever et al., 2014; Lutz et al., 2009; Pannasch et al., 2011) but not Cx30 alone (Pannasch et al., 2014) appear swollen, which suggests a mechanism for controlling the astrocyte volume and PAP displacement.

## Regulating Synaptic Morphogenesis Through PAPs?

Structural plasticity of PAPs might also have a direct effect on synaptic environment. In hippocampal slices, spines disappear more frequently when PAPs are not present (Nishida and Okabe, 2007). The same effect was found when ephrin signaling was perturbed, and dendritic protrusions disappeared faster, although they were in contact with astrocytic processes (Nishida and Okabe, 2007). Furthermore, blocking Rac‐1 and interfering with PAP motility leads to enhanced number of dendritic filopodia (Nishida and Okabe, 2007). Similarly, in the cerebellum Bergmann glia coverage is required for synapse stabilization and regulation (Lippman Bell et al., 2010; Lippman et al., 2008).

Indeed, astrocytes secret several factors that might play a role in such phenomena. Amongst the factors are cholesterol (Diniz et al., 2014; Mauch et al., 2001), leptin (Kim et al., 2014), transforming growth factor (TGF)‐β (Diniz et al., 2012, 2014), TNF‐α (Han et al., 2013) and several ECM molecules (Dityatev and Rusakov, 2011; Heikkinen et al., 2014; Jones and Bouvier, 2014; Risher and Eroglu, 2012; Thalhammer and Cingolani, 2014). The most intensely studied ECM molecules involved in synapse formation are the thrombospondins (Christopherson et al., 2005; Risher and Eroglu, 2012). These molecules induce the formation of synapses and the maturation of dendritic spines in several experimental settings (Christopherson et al., 2005; Eroglu et al., 2009; Garcia et al., 2010; Risher and Eroglu, 2012). Other astrocyte‐derived ECM molecules that are important in synaptogenesis are hevin and its antagonist the secreted protein acidic and rich in cysteine (SPARC) (Kucukdereli et al., 2011). Their fine‐tuned secretion allows astrocytes to dynamically adjust activity levels of synapses (Albrecht et al., 2012; Jones et al., 2011). Moreover, the heparan sulfate proteoglycans, glypican 4, and glypican 6 are involved in synapse formation (Allen et al., 2012). Also astrocyte‐derived chondroitin sulfate proteoglycans such as brevican, neurocan, and phosphacan are important for synapse maturation as they stabilize AMPARs at synapses (Allen, 2013; Frischknecht et al., 2009; Pyka et al., 2011). In addition, both neurons and astrocytes produce the components for the formation of perineuronal nets (PNNs) (Faissner et al., 2010). These ECM structures might have structural roles and help compartmentalizing the CNS as well as representing a diffusion barrier for neurotransmitter spillover just like PAPs do (Faissner et al., 2010; Frischknecht et al., 2009; Frischknecht and Seidenbecher, 2008). Molecular interactions between synapses, local astroglia and the extracellular matrix remain an intriguing and promising field for scientific exploration (Dityatev and Rusakov, 2011).

## Implications of PAP Morphogenesis for Synaptic Function

Astrocytic coverage of synapses is essential to limiting extrasynaptic glutamate escape and hence “undesirable” synaptic crosstalk. The heterogeneity of astrocytic coverage across different brain regions suggests, however, that glutamate spillover is favored in certain areas and suppressed in others (Bernardinelli et al., 2014a; Lange et al., 2012; Oliet et al., 2008). For example, in the supraoptic nucleus (SON), astrocytes undergo extensive morphological changes in lactating rats (Hatton, 1997; Piet et al., 2004a; Theodosis and Poulain, 1993). Astrocytic coverage is reduced and this leads to an enhanced number of synapses and neurotransmitter spillover (Hatton, 1997; Piet et al., 2004a; Theodosis and Poulain, 1993). With the reduction of astrocytic synapse enwrapping also glutamate clearance is reduced which in turn leads to enhanced negative feedback on glutamate release and increased heterosynaptic depression of GABA release (Oliet et al., 2001; Piet et al., 2004b) and prolonged excitatory postsynaptic potentials (EPSPs) in the cerebellum (Iino et al., 2001). Additionally, in such cases, less d‐serine may be available from astrocytes for the co‐agonist activation of postsynaptic NMDARs and thus the machinery of long‐term synaptic plasticity in the SON (Panatier et al., 2006). In the same area of the hypothalamus glial coverage of neurons also follows a circadian rhythm with changes of dendritic enwrapping during day and night (Becquet et al., 2008).

Detailed modeling of release events in the synaptic environment suggests that covering half of the synaptic perimeter by PAPs can reduce extra‐cleft glutamate transients several‐fold, and 95% coverage enhances this phenomenon by an order of magnitude (Rusakov, 2001; Zheng et al., 2008). Clearly, changes in PAP coverage have important consequences for glutamate escape and subsequent activation of perisynaptic glutamate receptors. Interestingly, high‐affinity receptors, such as NMDARs or mGluRs, which are located just outside the synaptic cleft, appear most sensitive to changes in extrasynaptic glutamate escape. This is because the glutamate concentration time course at that locus corresponds to the highest dynamic range of receptor responses (Sylantyev et al., 2013; Zheng et al., 2008). Thus, such receptors could be “strategically” positioned to respond strongly to any small changes in astroglial coverage of the synapse. Re‐arrangement of such a complex synaptic microenvironment during use‐dependent astroglial changes might also contribute to the differential action of two NMDARs co‐agonists, glycine and d‐serine, depending on the intra‐ or extrasynaptic receptor location (Papouin et al., 2012). Finally, it is logical to think that volumetric changes in perisynaptic astroglia are capable of physically altering the local environment, restricting or permitting molecular signal exchange among its various components.

## Monitoring of Astroglia on the Nanoscale: Emerging Techniques

The progress in astroglial research has been somewhat hampered by our current inability to monitor and probe, in real time, the organization and function of ultrathin processes that represent the bulk of astroglial morphology *in situ*. This is mainly because the size of such processes (50–200 nm) is beyond resolution of conventional optical microscopy (200–300 nm). Furthermore, so far no reliable marker exists to enable distinguishing astrocyte processes that contact synapses from those that do not (Reichenbach et al., 2010). At the same time, the widely diverse physiological actions attributed to astrocytes suggest the possibility of fine sub‐cellular compartmentalization inside individual cells (Rusakov et al., 2014): it cannot be excluded, for instance, that PAPs have a molecular identity distinct from other processes. The key to tackling these issues appears to lie in the emerging wave of super‐resolution imaging techniques that generally overcome limits of conventional light microscopy. A detailed consideration of various super‐resolution methods is outside the scope of this review (for recent surveys see (Fornasiero and Opazo, [Link]; Yamanaka et al., 2014; Zhong, [Link]); also *Table 1*). Instead, here, we will briefly discuss respective applications that could be relevant to studying astroglia.

**Table 1:** Super‐Resolution Fluorescence Microscopy

Experimental factor	PALM/STORM	SIM	STED
Key element to achieve super‐resolution	Photoactivation *cis‐trans* isomerization, triplet pumping, etc.	Structured illumination	Stimulated emission
Microscope type	Wide‐field	Wide‐field	Laser scanning
The number of required excitation light wavelengths	1–2	1	2
Spatial resolution in *x*‐*y* plane	10–30 nm	100–130 nm	20–70 nm
Spatial resolution in *z* direction	10–75 nm	∼300 nm	40–150 nm
	Using opposing lenses, optical astigmatism, dual focus imaging or double‐helical PSF		Using two opposing lenses or *z*‐phase mask
*Z*‐stack range	Few hundred nm–few µm	∼few µm	∼20 µm
Frame rate	s–min	ms–s	ms–s
Applicable fluorescent probe	Photoswitchable fluorescent proteins/molecules	Any if photostable	Any if photostable
Photodamage	Low–moderate	Moderate	Moderate–high
Photobleaching	Low	Moderate–high	Moderate–high
Required postimage processing	Yes	Yes	No

Adapted from (Yamanaka et al., 2014).

**Figure 5:**
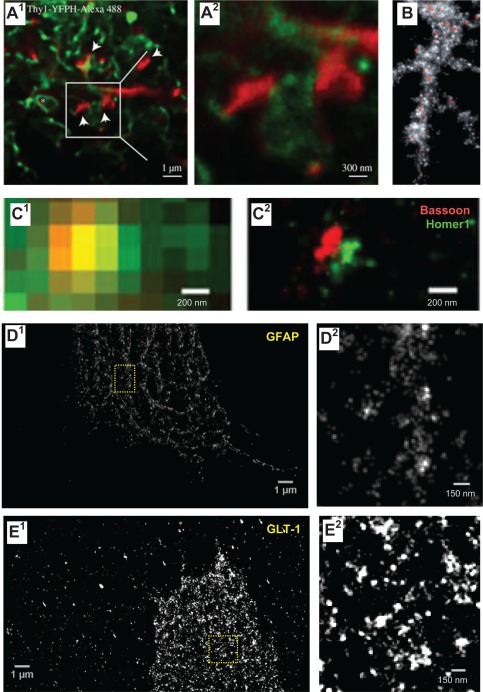
Advancing super‐resolution microscopy for astroglial research. (**A**) STED images showing CA1 stratum radiatum astrocytic processes (green, whole‐cell loading with Alexa Fluor 488) adjacent to synaptic structures in organotypic slices (red; Thy1‐YFP; dendritic spines, arrows) at lower (A1) and higher (A2, area indicted by square in A1) magnification; asterisk, O‐ring structures indicating tentative cell process envelopes; adapted from (Panatier et al., 2014). (**B**) STED image of P2Y1 receptors (red) along a multi‐branched astrocytic process (glutamine synthase, grey) in the adult mouse hippocampus. Adapted from (Volterra et al., 2014). (**C**) STORM imaging of pre‐ (Bassoon; red) and postsynaptic (Homer1; green) scaffolding proteins in the mouse main olfactory bulb glomeruli imaged using conventional fluorescence imaging (C1) and STORM (C2); adapted from (Dani et al., 2010). (**D**, **E**) dSTORM images of cultured (14 DIV) mixed glial cells from rat hippocampus (ProLong Diamond in Zeiss Elyra PS.1 microscope; Fiji Plugin ThunderSTORM, 3,000 frames); unpublished data by J. Heller. (D) GFAP stained with monoclonal antibody (Novus, GA5, secondary Alexa Fluor 488 donkey anti‐mouse antibody, Life Technologies) shown at lower (D1) and higher (D2, fragment indicated in D1) magnification. (E) GLT‐1 (polyclonal, Millipore) visualized with Alexa Fluor 568 goat anti‐guinea pig antibody (Life Technologies) shown at lower (E1) and higher (E2, fragment indicated in E1) magnification. Note that the cells were permeabilized, and therefore GLT‐1 was stained throughout cellular compartments.

Stimulated‐emission depletion (STED) microscopy uses conventional excitation which is partly quenched (depleted) at its periphery by the doughnut‐shaped focal illumination: this reduces the emission spot thus boosting resolution beyond the diffraction limit (Klar et al., 2000). The STED methodology has successfully been used to monitor the fine structure of dendritic spines (Bethge et al., 2013; Ding et al., 2009; Tonnesen et al., 2014, 2011), and it has recently been applied to image astroglia (Panatier et al., 2014) (Fig. 5A). It appears that STED imaging could also reveal the heterogeneous distribution of purinergic P2Y1 receptors along astrocyte processes (Volterra et al., 2014) (Fig. 5B). Whilst STED imaging conveniently provides super‐resolution with acquisition protocols common for more conventional imaging modes (such as two‐photon excitation imaging and photolytic uncaging), this is achieved at the expense of much higher laser power applied to the preparation, and higher equipment costs.

Another set of super‐resolution techniques concerns single‐molecule localization imaging, such as photo‐activated localization microscopy (PALM) or stochastic optical reconstruction microscopy (STORM), which are based on reconstructing the point source of fluorescence (often one molecule) from its detected image which is “blurred” by diffraction (Betzig et al., 2006). These techniques require stochastic excitation of only a small (sparsely distributed) fraction of fluorescent molecules per imaging cycle. Repeating such cycles thus accumulates exact positions for most or all fluorescent molecules in the field of view. Although this type of approach could provide excellent spatial resolution (Table 1), its use in organised live tissue is limited, mainly because either the imaging conditions or the probe chemistry are not compatible with physiology. Nonetheless, there have been some spectacular revelations regarding the nanoscopic distribution of pre‐ and postsynaptic receptor proteins (Dani et al., 2010) documented in fixed tissue (Fig. 5C). Furthermore, our attempts to visualize astroglial protein distribution using direct STORM (dSTORM) provide evidence for the technical plausibility of this approach (Fig. 5D,E).

A general super‐resolution approach conceptually related to PALM enables tracking of individual molecules conjugated to bright nanoparticles, such as quantum dots, in live cells (reviewed in (Medintz et al., 2005)). This technique achieves point‐source fluorescence recording, and thus nanoscale localization, by registering rare intermittent “blinking” (random switching) of individual nanoparticles. Single‐particle tracking (and related PALM approaches) does not require high‐powered switching or quenching and has been successfully adapted for neuronal culture preparations (and very recently for organotypic brain slices (Biermann et al., 2014)). Over the years it has helped to uncover fundamental features of synapses pertinent to their dynamic organization on the nanoscale (recently reviewed in (Choquet and Triller, 2013)). However, application of such methods to astroglia is only beginning to emerge. Single‐molecule tracking of mGluRs in cultured astroglia has provided some previously unattainable insights into the molecular dynamic organization of astrocytic compartments (Arizono et al., 2014, 2012; Shrivastava et al., 2013). Most recently, a similar method has unveiled strong, activity‐dependent mobility of glutamate transporters GLT‐1 on the surface of cultured astroglia (Murphy‐Royal et al., 2015), thus opening a new horizon in our understanding of astroglial microphysiology.

## Concluding Remarks

A growing body of experimental evidence suggests that astrocyte signaling can, at least in certain conditions, influence excitatory synaptic transmission and its use‐dependent plasticity in a Ca^2+^‐dependent manner (Henneberger et al., 2010; Jourdain et al., 2007; Min and Nevian, 2012; Navarrete et al., 2012; Parri et al., 2001; Perea and Araque, 2007). This does not necessarily imply that elegant experimental manipulations with astroglial Ca^2+^ within a certain dynamic range by triggering certain cellular cascades should reproduce such effects (Agulhon et al., 2010; Fiacco et al., 2007; Petravicz et al., 2008) (see (Rusakov et al., 2014; Volterra et al., 2014) for discussion). In addition to the much debated astrocyte‐neuron exchange, Ca^2+^ rises in astrocytes could also boost the expression level of glutamate transporters (Devaraju et al., 2013), re‐position mitochondria closer to glutamate transporters (Jackson et al., 2014; Ugbode et al., 2014), and regulate neuro‐metabolic coupling with neurons (Bernardinelli et al., 2004; Porras et al., 2008). Recent findings suggest that such Ca^2+^ signals could be required for morphological changes in PAPs (Molotkov et al., 2013; Tanaka et al., 2013).

The PAP remodeling, with or without local rearrangement of glutamate transporter proteins, is capable of altering the efficiency of extrasynaptic glutamate clearance or escape (Benediktsson et al., 2012; Danbolt, 2001; Panatier et al., 2006; Zheng et al., 2008) (although (Diamond et al., 1998)) and hence affecting the modus operandi for local excitatory circuitry. Altered glial coverage could also affect astrocyte‐neuron lactate transport contributing to synaptic efficacy (Suzuki et al., 2011) and local water homeostasis (Simard and Nedergaard, 2004). In summary, there is a plethora of physiologically plausible candidate mechanisms that could underpin the adaptive role of astroglial remodeling in local neural network activity. To date, however, the bulk of experimental evidence to that effect has been obtained using either conventional optical microscopy (including confocal and two‐photon excitation fluorescence approaches) in live cells or using EM in fixed tissue. Although such evidence has been essential in advancing our understanding of astroglia‐neuron communication, it leaves unresolved some critical questions regarding molecular physiology of the synaptic microenvironment, and in particular, local astroglial protrusions, on the nanoscale. Single‐molecule tracking of mGluRs and glutamate transporters in cultured astroglia has provided first glimpses on what could be the dynamic molecular micro‐organization of astrocytes (Arizono et al., 2012; Murphy‐Royal et al., 2015; Shrivastava et al., 2013), and STED imaging in organised brain tissue (Tonnesen et al., 2014) has opened up the nanoscopic world of live astroglial architecture *in situ*. Clearly, further implementation of super‐resolution techniques in organised brain tissue should help to bridge the knowledge gap in our understanding of the molecular micro‐physiology of astroglia and its role in information processing of brain networks.
